# Young children have limited mucosal immunity when stimulated with an influenza vaccine in tonsil organoids

**DOI:** 10.21203/rs.3.rs-7842732/v1

**Published:** 2025-10-30

**Authors:** Mark Davis, Meng Sun, Elsa Solà, Jing Guo, John Valainis, Vishnu Shankar, Lilit Kamalyan, Neha Gupta, Mustafa Ghanizada, Christian Constantz, Vamsee Mallajosyula, Azam Mohsin, Xiaorui Han, Robson Capasso

**Affiliations:** Stanford University School of Medicine; Stanford University School of Medicine; Institute for Immunity, Transplantation and Infection, Stanford University School of Medicine, Stanford, California 94305; Stanford University; Institute for Immunity, Transplantation and Infection, Stanford University School of Medicine; Stanford University; Institute for Immunity, Transplantation and Infection, Stanford University School of Medicine; Stanford University School of Medicine; Stanford University School of Medicine; Center for Infectious Medicine, Department of Medicine Huddinge, Karolinska Institutet; Stanford University; Stanford University School of Medicine; Stanford University School of Medicine; Stanford University

## Abstract

While it has been known for many years that children under five years old are much more vulnerable to most infectious diseases than older children or adults, we know very little about the specific immunological reasons. Thus, we leveraged our recently developed tonsil organoid model, a high-resolution in vitro system of human immunity to vaccination, to compare tonsils from children as young as 2 years old to those from young adults. After stimulation with the live attenuated influenza vaccine, toddlers exhibited lower levels of influenza-specific IgA and IgG antibodies, limited T-independent response, and fewer activated cytotoxic CD8+T cells, all critical components supporting influenza defense. Additionally, toddlers showed reduced levels of key cytokine signaling proteins, including FLT3L, IL2, IL17, TACI, which are important in antibody class switching. Conversely, toddlers produced more of the pro-inflammatory cytokines CCL2 and PAI1, both associated with more severe influenza infection. We observed fewer interactions between T and B cells and diminished TLR and T-bet signaling in toddlers than in adults. Further analysis identified distinct metabolic disadvantages in toddlers, particularly within germinal centers, observed in a time-dependent manner. Machine learning analyses of our multi-omic data highlighted dominant variables and key predictors that distinguish diverse immune responses among groups. Our study used systems approaches to underscore critical deficits in cellular compositions, cytokine profiles, intracellular signaling, cell-cell interactions, and metabolic programs in young children’s immune systems under vaccine/viral stimulation, offering valuable guidance for future vaccine development and therapies.

## Introduction

Globally, millions of people die from respiratory infections every year, with young children and the elderly being the most vulnerable^1^. This trend was even more acute before the widespread introduction of childhood vaccine, antibiotics and modern sanitation, when almost half of children under five died of infections. But our knowledge of early childhood immunity has been very limited because clinical studies are difficult and blood volumes are very small. It is known that the differences are significant, such that vaccine trials are conducted separately in young children and adults due to significant differences in immune function between these age groups. Recently we developed an in vitro model to study vaccine responses in organoids derived from tonsils^2^, which is part of the mucosal immune system, and where we were able to observe many of the hallmarks of adaptive immunity, such as germinal center formation, specific T and B cell mobilization, antibody secretion, class switching and affinity maturation^3^. These tonsils are derived from children as young as 2 years of age, and adults as old as 70 years or more. In both age groups tonsillectomies were performed to relieve sleep apnea. In this study, consistent with clinical data^4–7^, we found that T-independent (TI) B cell responses are very poor or absent in young children, and only become evident in children over five years of age^2,4^. Here we seek to follow up on that observation by using single-cell technology to comprehensively analyze the immune responses of toddlers under five, older children and young adults using the tonsil organoid system stimulated with the live attenuated influenza vaccine (LAIV). This is an intranasal vaccine that mimics the natural infection route, eliciting both mucosal and systemic immune responses in mucosa-associated lymphoid tissues. In contrast, inactivated influenza vaccine (IIV), which is administered parenterally, induces a poor mucosal response^8^. Mucosal vaccines protect by preventing pathogens from entering hosts at barriers, whereas injectable vaccines primarily curtail infections and reduce symptoms^9^. Despite the benefits of intranasal administration for protecting against respiratory infections, challenges remain, including incomplete knowledge of protective mucosal immune responses and the lack of approved mucosal adjuvants. To date, only LAIV has been FDA-approved, though several other candidates targeting respiratory pathogens, including COVID-19, are currently in clinical trials^10^.

Multi-omic systemic approaches combined with machine learning analyses, including an AI foundation model, revealed striking differences in the immune responses of adults vs these young children. Overall, our study provides a roadmap for understanding how the less developed immune systems of young children respond to influenza vaccination, with significant implications for improving therapeutic strategies and vaccine designs for infectious diseases.

## Results

### Toddlers exhibit limited mucosal humoral response to LAIV in tonsil organoids.

**Figure 1:**
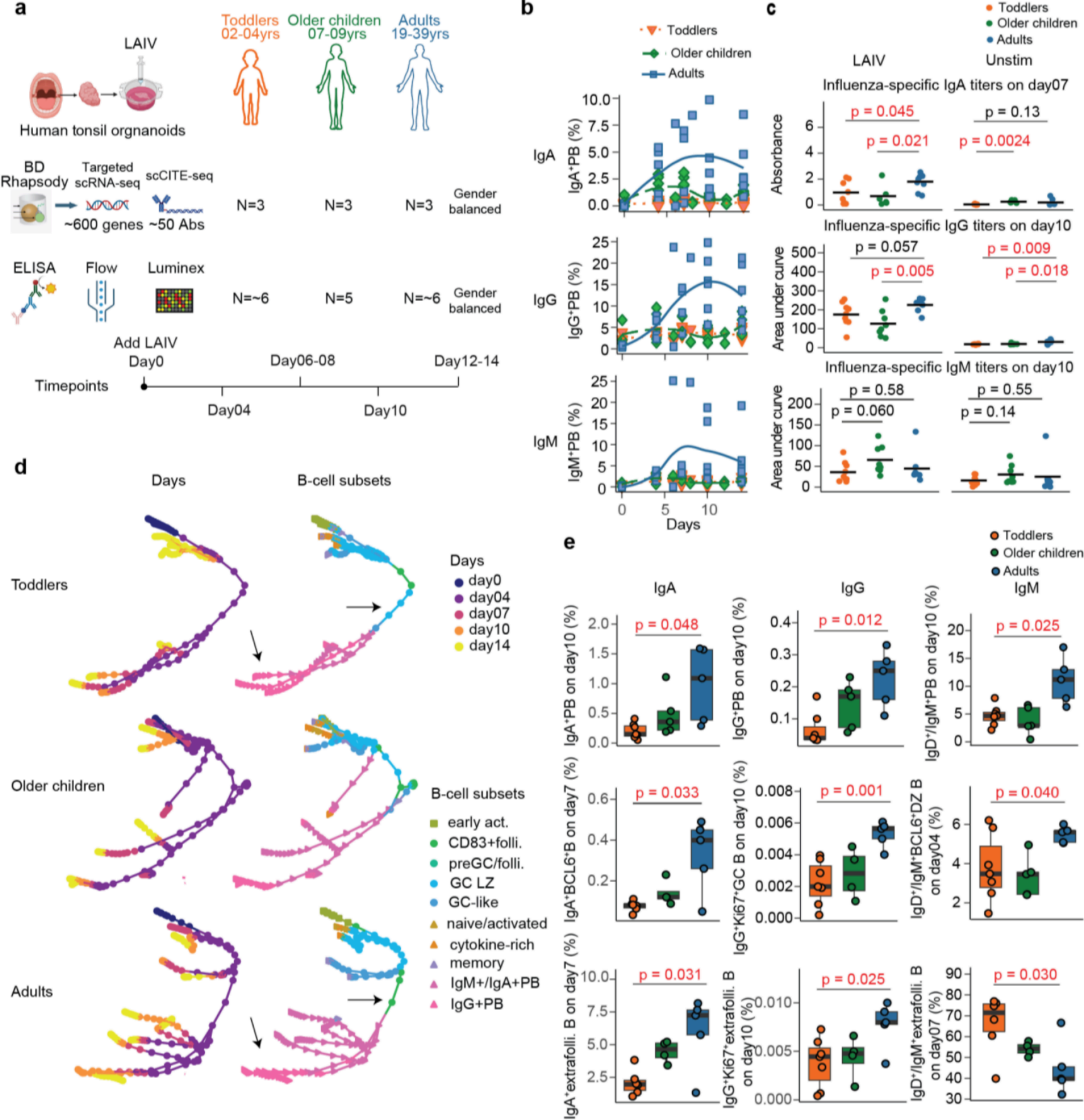
Toddlers exhibit limited humoral response to LAIV in tonsil organoids. (**a**) Our study investigated age-related differences in the human immune response to LAIV using tonsil organoids from donors across various age groups. Initial findings were obtained through scRNA-seq using the BD Rhapsody platform and further validated in a larger number of donors using ELISA, flow cytometry and Luminex assays. (**b**) scRNA-seq data revealed that toddlers had lower levels of IgA^+^ and IgG^+^PBs in B cells compared to adults. (**c**) ELISA assays demonstrated lower levels of Influenza-specific IgA and IgG titers in toddlers relative to adults. (**d**) Time trajectory analysis from scRNA-seq data highlighted differences in B-cell differentiation lineages across age groups, particularly in class-switched PB lineages and their precursors, as indicated by arrows. (**e**) Flow cytometry validated reduced fractions of PBs, GC and extrafollicular B cell subsets across all isotypes in B cells in toddlers than adults. Statistical significance in **c** and **e** was calculated using the two-sided Student’s t-tests.

To study the impact of age on immune responses to LAIV, we divided donors into three age groups: toddlers (2–4 yrs), older children (7–9 yrs), and adults (19–39yrs) (Supp. Table 1). We found that approximately 45% of the toddlers and 67% of the adults tested produced influenza-specific pan-Ig (IgM, IgA and IgG) antibodies exclusively in the LAIV-stimulated samples (Extended Data Fig.1a), similar to previously reported findings^2^, but with an increased fraction as age increased. Among the LAIV-responsive donors, we closely monitored and characterized their immune responses in tonsil organoids at multiple time points: around baseline (day0), day04, day07, day10 and day14 post-LAIV stimulation, employing multi-omic approaches including high-throughput single-cell RNA sequencing (scRNA-seq) and CITE-seq via the BD Rhapsody platform, ELISA, flow cytometry and Luminex assays (Fig. 1a).

In the scRNA-seq with CITE-seq analysis targeting approximately 600 genes and 50 surface proteins that are associated with key immune activities, we observed reduced plasmablast (PB) enrichment across all three isotypes--IgM, IgA, and IgG--in toddlers compared to adults following LAIV stimulation (Fig. 1b and Extended Data Fig.1b-d). Notably, *IGHA1*^+^PB cells showed more than a 10-fold enrichment in adults compared to toddlers, while *IGHG2*^+^PB cells exhibited a 5-fold enrichment in adults (Extended Data Fig.1b). Interestingly, *IGHG2*^+^PB cells were predominantly enriched only in adults, whereas *IgA1*^+^PB cells were enriched in both older children and adults relative to toddlers. This observation aligns with previous findings that IgG2^+^B cells and their associated anti-carbohydrate antibodies develop later in childhood^11^. These PB findings were supported by ELISA assays, which measured influenza-specific antibody titers and revealed lower antibody levels in toddlers, particularly for class-switched antibodies IgA and IgG (Fig. 1c). Given the critical role of IgA and IgG in humoral protection, we conducted trajectory analysis on the scRNA-seq data to further investigate PB and antibody generation process. This analysis showed that toddlers exhibited fewer PB lineages and reduced isotype diversity compared to adults (Fig. 1d). Additionally, in toddlers, CD83^+^follicular B cells, GC light zone (LZ), and GC-like B cells acted as PB precursors, whereas in adults, CD83^+^follicular B cells directly differentiated into PBs (as highlighted by arrows in Fig. 1d). Furthermore, scRNA-seq data revealed that toddlers had lower frequencies of memory and GC-like B cells, along with a higher proportion of naïve B cells (Extended Data Fig.1d).

To further validate the differences in B cell subsets potentially contributing to the limited humoral responses in toddlers, we performed flow cytometry, which confirmed reduced PB and GC activities across all isotypes in toddlers relative to adults (Fig. 1e). Given that both GC and extrafollicular B cells can differentiate into PBs with distinct timelines and degrees of affinity maturation, we also observed lower fractions of extrafollicular B cells expressing class-switched isotypes in toddlers, potentially contributing to their reduced PB differentiation (Fig 1e). Notably, IgA^+^BCL6^+^B cells showed a 4-fold increase in adults compared to toddlers, while IgA^+^extrafollicular B cells exhibited a 3-fold difference. These findings highlight potential key developmental differences in B cell dynamics that underlie the limited humoral responses observed in toddlers.

### Toddlers exhibit limited class switch-related cytokines.

**Figure 2:**
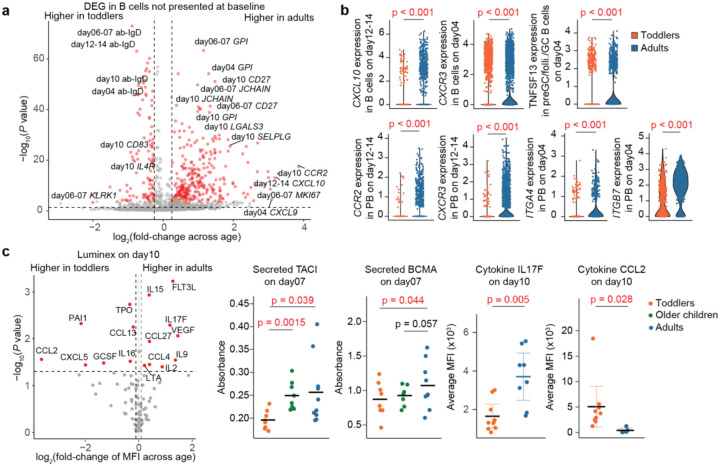
Toddlers exhibit limited cytokine profiles related to CSR in response to LAIV. (**a**) A volcano plot showing DEGs in B cells from scRNA-seq data between toddlers and adults at multiple timepoints after LAIV stimulation. The fold-changes represent the ratio of the mean log-normalized expression levels between the adult and toddler groups. A positive log_2_(fold-change) indicates higher mean expression levels in adults compared to toddlers. Red, B-cell subsets with P value < 0.05. ab- represents antibody signals from CITE-seq. (**b**) Violin plots displaying the log-normalized expression levels of representative DEGs in B cell subsets among toddlers and adults as in **a.** (**c**) Luminex and ELISA assays confirming lower levels of cytokines and secretory cytokine receptors related to CSR in toddlers compared to adults. Statistical significance in **a** and **b** was calculated using two-sided Wilcoxon rank-sum tests and adjusted by the false discovery rate method. Statistical significance in **c** was determined by two-sided Student’s t-tests.

To investigate the underlying reasons for the limited humoral responses observed in toddlers, we analyzed differentially expressed genes (DEGs) in B cells from the scRNA-seq data (Fig. 2a). Our analysis revealed that toddlers showed the most significant downregulation of genes encoding cytokines and their receptors, including *CCR2* and *CXCL10* (Fig. 2b and Supp. Table 2). Given the crucial role of cytokine signaling in immune function, these reductions may contribute to the limited humoral responses observed in toddlers. To validate these findings, we profiled cytokines in tonsil organoid supernatants using Luminex assays. This analysis revealed significantly lower levels of class switch recombination (CSR)-related cytokines, such as FLT3L, IL15, and IL2, in toddlers compared to adults (Fig. 2c). Additionally, IL17F, a cytokine associated with mucosal protection, was also reduced in toddlers, though its role in viral protection remains unclear^12^. Conversely, toddlers exhibited elevated levels of CCL2 and PAI1, which are associated with increased severity of influenza infection and inflammation^13,14^. Cytokine module analysis of the Luminex data further confirmed the downregulation of modules containing IL15, CXCL9/10, CCL4/5, VEGFA, and TNFSF10 in toddlers, while CXCL13 module was upregulated (Extended Data Fig.2a and Supp. Table 3), consistent with the scRNA-seq results.

We also examined additional cytokines related to B-cell differentiation, including BAFF and its homolog APRIL, which were not included in the Luminex panel. The scRNA-seq data revealed significantly lower expression of *TNFSF13* (APRIL) in toddlers, particularly in non-memory B cells (Fig. 2b and Extended Data Fig.2b). ELISA assays showed negligible levels of BAFF and APRIL in supernatants, likely due to receptor binding including TACI and BCMA (as stated in the product manuals), which could obscure ELISA detection. Instead, we measured the soluble forms of these receptors in supernatants and found lower levels of them in toddlers, particularly lower levels of secretory TACI compared to older children and adults (Fig. 2c). Dysfunction of TACI is associated with IgA deficiency^15^ and impaired T-independent responses^16^, which likely contribute to the reduced IgG2^+^PB observed earlier. ELISA assays showed no differences in active or total TGF-b1 levels between toddlers and adults (data not shown). These findings suggest that limited signaling through BCMA, TACI, and key cytokines such as IL2, IL15, and IL17F likely contributes to the reduced humoral and CSR responses in toddlers.

Nasopharyngeal-associated lymphoid tissues play critical roles not only in first-line pathogen defense but also in training pathogen-specific immune cells to home to respiratory tracts and lungs. We observed lower expression of lung-homing chemokine receptors^17^, such as *CCR2* and *CXCR3*, in B cells from toddlers compared to adults, particularly within the PB subset (Fig. 2b and Supp. Table 2). This suggests a reduced migratory capacity of antibody-secreting B cells to other tissues in toddlers. Additionally, toddlers’ B cells exhibited lower levels of integrins *ITGA4* and *ITGB7*, especially in PBs (Fig. 2b and Supp. Table 2), which are critical for interactions with high endothelial venules (HEV) to facilitate trafficking through mucosal-associated lymphoid tissues^18^. Gene regulatory network analysis^19^ revealed that these homing receptors were predominantly regulated by *TBX21* (T-bet) (Extended Data Fig.2c). Further gene module analysis based on co-expression to infer regulatory networks also showed weaker T-bet signaling in toddlers (Extended Data Fig.3a), reflecting impaired anti-viral-related CSR activities and effector B-cell functions in response to cytokines^20^ among toddlers.

To investigate the age-related differences in gene modules further, we analyzed their interactions using partial correlation networks (Extended Data Fig.3b and Supp. Table 3). The most pronounced interaction in adults, compared to toddlers, based on the fold-change in interaction strength, was between the PB and proliferation modules. Additionally, adults exhibited significant interactions between the naïve module and both the *TLR7/MYC* and *BCL6* signaling modules, interactions that were notably absent in toddlers. The absence of these interactions in toddlers may contribute to their poor T-independent (TI) B cell responses, as TLR7 serves as a key innate pattern-recognition receptor for TI influenza antigens. TLR stimulation can upregulate TACI^16^, a critical cytokine receptor required for TI responses, thereby promoting the generation of IgG2^+^PB we observed earlier. Interestingly, some interactions enriched in adults on day 7 were not detectable at baseline. These included interactions between the type I IFN and T-bet modules, the naïve and BCL6 modules, the naïve and proliferation modules, the TACI/CSR and PB modules, and the TACI/CSR and proliferation modules. This suggests that these age-specific enhancements in gene module interactions were triggered by LAIV stimulation. For interactions enriched in adults at both baseline and day 7, the degree of enrichment was significantly stronger on day 7, underscoring the impact of LAIV stimulation in amplifying these age-related differences. Collectively, these findings provide key insights into the developmental and molecular mechanisms underlying the limited humoral responses observed in toddlers.

### Toddlers have limited T-cell activity including cytotoxicity.

**Figure 3:**
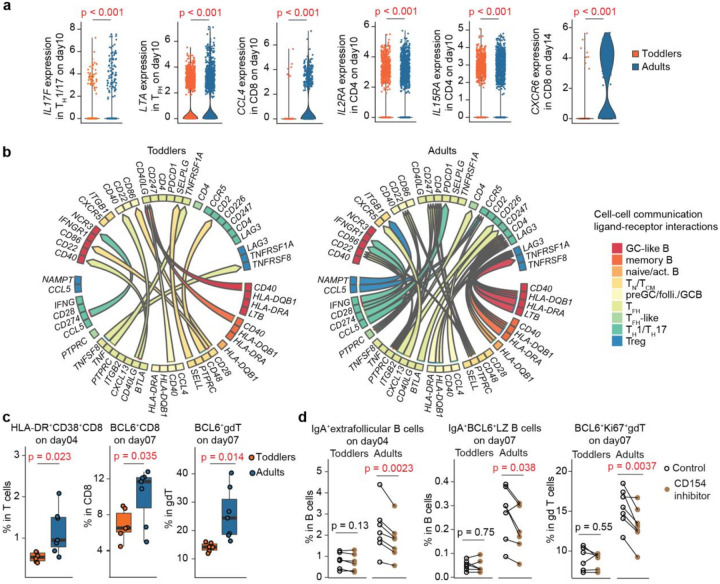
Toddlers had limited T-cell activities including cytotoxicity. (**a**) Violin plots illustrating log-normalized expression levels of DEGs in T cell subsets among toddlers and adults from scRNA-seq data. (**b**) Network plots showing estimated cell-cell communication interactions among B and CD4 T cell subsets in toddlers and adults based on their DEG as in **a**. (**c**) Flow cytometry revealing that activated CD8 (HLA-DR^+^CD38^+^), follicular CD8 (BCL6^+^), and follicular gdT (BCL6^+^) cells were less pronounced in toddlers than adults. (**d**) Flow cytometry showing that CD154 inhibition induced changes in IgA^+^extrafollicular (BCL6^−^) B, IgA^+^follicular (BCL6^+^) LZ (CD83^+^CXCR4^−^) B, and proliferating (Ki67^+^) follicular (BCL6^+^) gdT cells. Statistical significance in **a** was calculated using two-sided Wilcoxon rank-sum tests and adjusted by the false discovery rate method. Statistical significance in **c** and **d** was determined by two-sided Student’s t-tests.

In our investigation of age-related cytokine profiles, we noticed that certain critical cytokines, such as IL2 and IL17F, which were secreted at lower levels in toddlers, are reported to be primarily produced by T cells^21,22^. To further explore this, we analyzed DEGs from T-cell scRNA-seq data (Extended Data Fig.4a and Supp. Table 4) and found lower cytokine expression in T cell subsets, particularly *IL17F, LTA*, and *CCL4* in CD4 T_H_1/T_H_17, T_FH_ or CD8 T cells, in toddlers compared to adults (Fig. 3a and Extended Data Fig.4b). Additionally, we observed lower expression of cytokine receptors in CD4^+^T cells, such as *IL2RA* and *IL15RA*, indicating limited cytokine signaling (Fig. 3a). Consistent with previous studies^23,24^, we observed a Th2-skewed phenotype in toddlers, characterized by elevated levels of *GATA3* expression (Supp. Table 4). In CD8^+^T cells, adults exhibited higher levels of *CXCR6* (Fig. 3a), a marker associated with tissue-resident T cells that expand in response to IL15 and possess enhanced lung infiltration capabilities^25^. To better understand the B cell-CD4^+^T cell interactions, we employed MultiNicheNet, a cell-cell communication analysis tool^26,27^, by inferring ligand and receptor interactions from downstream target genes using DEGs. This analysis revealed that toddlers exhibited limited cytokine-receptor interactions, such as *CCL4-CCR5*, and cell antigen signaling, such as *CD22-PTPRC* and *HLA-DRCD4/LAG3*, but showed stronger *CD40-CD40LG* interactions compared to adults (Fig. 3b). The B-T cell interactions in toddlers appeared more restricted and less complex, possibly reflecting a less mature immune system compared to the more diverse interactions observed in adults.

Flow cytometry further confirmed lower levels of activated CD8, follicular CD8, and follicular gdT cells (Fig. 3c), which are crucial for cytotoxic responses to eliminate virus-infected cells and potentially play important roles in regulating follicular immune responses. Conversely, we observed a higher fraction of activated CD4^+^T cells, activated T_reg_, and total gdT cells in toddlers compared to adults (Extended Data Fig.4c), with T_reg_ potentially mitigating harmful inflammatory responses and dampening vaccine responses in toddlers^28,29^. Elevated T_reg_ levels were consistent with increased IL10 expression in both T and B cells, as observed in the scRNA-seq data (Supp. Table 2 and 4), along with a higher IL10 trend detected by Luminex (Supp. Table 3) in toddlers compared to adults. To investigate T-B cell interactions in more detail, we applied a CD154 (*CD40LG*) inhibitor, anti-CD154 antibody^30^, to disrupt these interactions in the organoids. Although cell-cell communication analysis predicted more robust *CD40-CD40LG* interactions in toddlers than in adults, the CD154 inhibitor induced less pronounced changes in toddlers (Extended Data Fig.4d). In adults, but not in toddlers, there was a noticeable decline in proliferating follicular gdT cells following CD154 inhibition (Fig. 3d). Similarly, the activities of IgA^+^B cells within and outside follicles decreased in adults after CD154 inhibition, a phenomenon not observed in toddlers (Fig. 3d). Both age groups showed a reduction in IgA^+^B cells and IgA^+^AID^+^B cells post-inhibition, but the effect was much stronger in adults (Extended Data Fig.4e). This greater impact in adults may result from a feedback mechanism involving B-T cell interactions beyond *CD40-CD40LG* signaling, such as those mediated by cytokines

### Toddler B cells exhibit limited metabolic activity.

**Figure 4:**
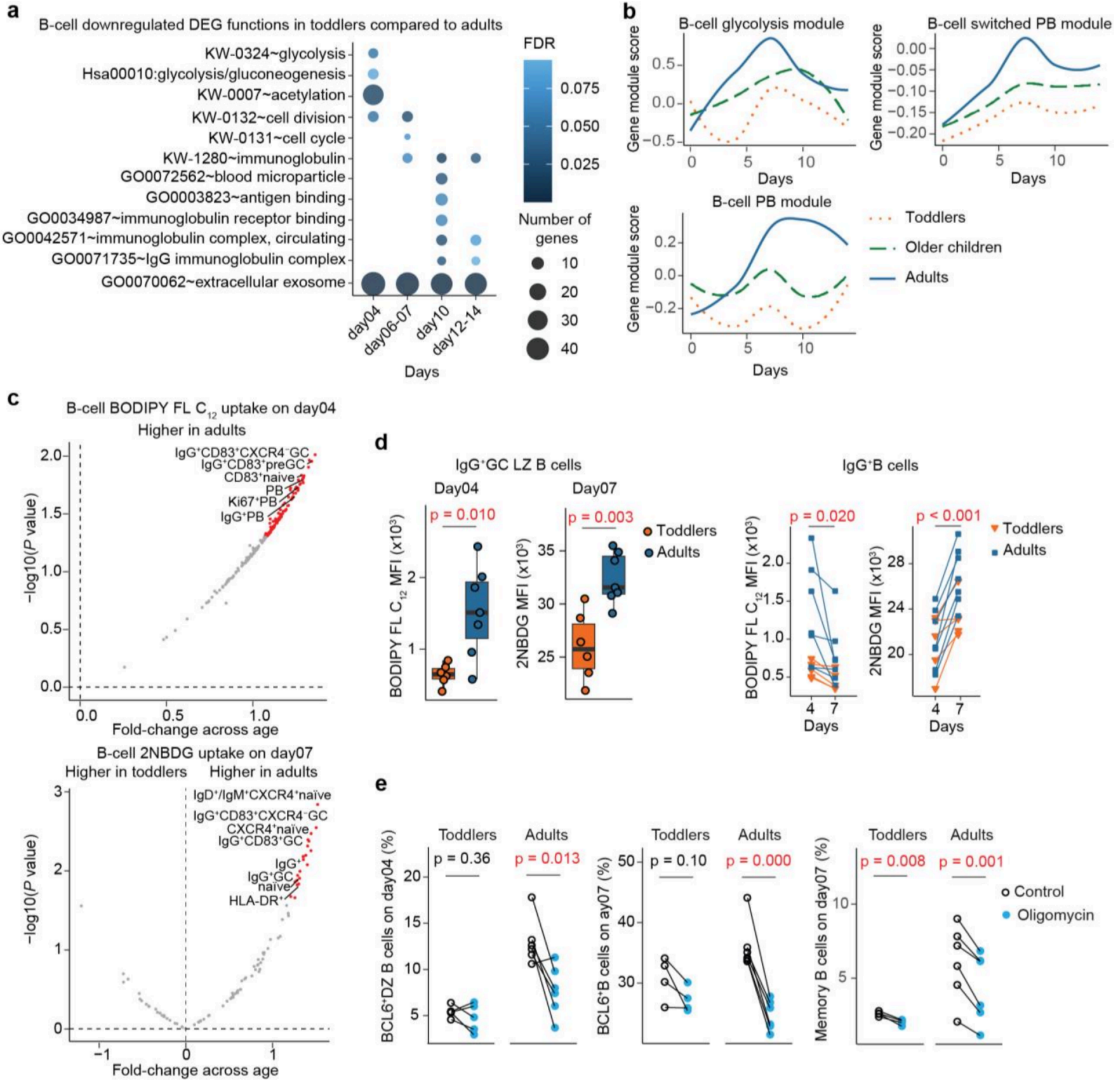
Toddlers exhibit limited metabolic activity. (**a**) Gene function enrichment analysis revealed downregulated B-cell functions in toddlers compared to adults based on DEGs from scRNA-seq data. The gene function reference used were UniprotKB Keywords (KW), KEGG pathways (hsa), and Gene Ontology (GO) databases. The false discovery rate (FDR) was calculated by a modified Fisher’s exact test with FDR correction. (**b**) Gene module analysis displayed distinct dynamics of co-expressed genes, peaking at various time points, based on scRNA-seq data. The dynamics of these gene modules were visualized by connecting the averaged gene module scores across all donors within each age group at multiple time points using a smooth function. (**c**) Volcano plots showed differences in cellular metabolite mean fluorescence intensities (MFI) from flow cytometry between toddlers and adults. The fold changes represent the ratio of metabolite MFI differences between adults and toddlers, normalized by their standard deviation. A positive fold change indicates higher cellular metabolite MFI levels in adults relative to toddlers. Red, B-cell subsets with P value < 0.05. (**d**) Plots illustrating BODIPY FL C_12_ and 2NBDG uptake in IgG^+^GC LZ (CD83^+^CXCR4^−^) and IgG^+^B cells. (**e**) OXPHOS inhibition by oligomycin induced reductions in BCL6^+^DZ (CXCR4^+^CD83^−^), BCL6^+^ and memory (CD27^+^CD38^−^) B cells, as measured by flow cytometry. Statistical significance in **c,d** and **e** was calculated using two-sided Student’s t-tests.

To investigate the functional differences in B cells across age groups, we analyzed DEGs using the Database for Annotation, Visualization and Integrated Discovery (DAVID)^31^ (Fig. 4a). Our findings indicated that adults displayed progressively increased activities in glycolysis, proliferation, and immunoglobulin production over time compared to toddlers. This suggests a typical immune sequence driven by metabolic needs, cell division, and antibody production in our organoid model. We also examined co-expressed gene modules and found lower glycolysis and PB activities in toddlers than in adults (Fig. 4b and Extended Data Fig.3b). Specifically, the rate limiting enzymes of glycolysis^32,33^, *PFKP* and *LDHA*, were expressed at lower levels in B cells (Supp. Table 2). Notably, we also observed reduced expression of the glutamine transporter *SLC1A5* in toddlers (Supp. Table 2), suggesting lower amino acid biosynthesis and limited OXPHOS activity, as glutamine supports this pathway^34^.

To validate the reduced metabolic activities in toddlers, we conducted additional experiments to thoroughly investigate age-related metabolic shifts. We assessed cellular uptake of glucose and fatty acids using a florescent glucose probe, 2NBDG (2-deoxy- 2-[(7-nitro-2,1,3-benzoxadiazol-4-yl)amino]-d-glucose), and a florescent lipid probe, BODIPY FL C_12_ (4,4-Difluoro-5,7-Dimethyl-4-Bora-3a,4a-Diaza-*s*-Indacene-3-Dodecanoic Acid)^35,36^. Flow cytometry revealed that glucose uptake was lower in toddlers than in adults on day07, particularly in naïve B cells, antigen-presenting (HLA-DR^+^) B cells, IgG^+^B cells, and IgG^+^GC B cells (Fig. 4c). Interestingly, we also observed consistent reductions in fatty acid uptake across all B cell subsets in toddlers compared to adults, with the most pronounced differences observed on day 4 (Fig. 4c and Extended Data Fig.5a). These reductions in both metabolites in toddlers may be linked to the high energy and nutrient demand required for growth^37^, which, in turn, can constrain immune activation and class-switched antibody production^38,39^.

Some B-cell subsets, such as IgG^+^GC LZ B cells, exhibited enhanced fatty acid uptake on day 4 and increased glucose uptake on day 7 in adults compared to toddlers (Fig. 4d). This suggests a metabolic transition from fatty acid to glucose uptake in these GC B cells during organoid culture. To further explore this, we demonstrated that certain B cell subsets, particularly IgG^+^B cell subsets, showed reduced fatty acid uptake and increased glucose uptake, reflecting a transition in metabolite preference over time (Fig. 4d and Extended Data Fig.5b).

While glucose and fatty acid uptake do not fully capture OXPHOS activity, we further assessed these changes by treatment with OXPHOS inhibitor Oligomycin A to our organoids. Inhibition of OXPHOS led to a more substantial reduction in GC and memory B cells in adults compared to toddlers (Fig. 4e and Extended Data Fig.5c). This finding suggests a greater dependence on OXPHOS for the development and maintenance of these cell subsets in adults compared to toddlers. Overall, our data demonstrated an increase in metabolic activities including glucose and fatty acid uptake, as well as OXPHOS activity, particularly within germinal centers, as individuals maturing from early childhood to adulthood.

### Age as the primary factor influencing immune response variation.

**Figure 5:**
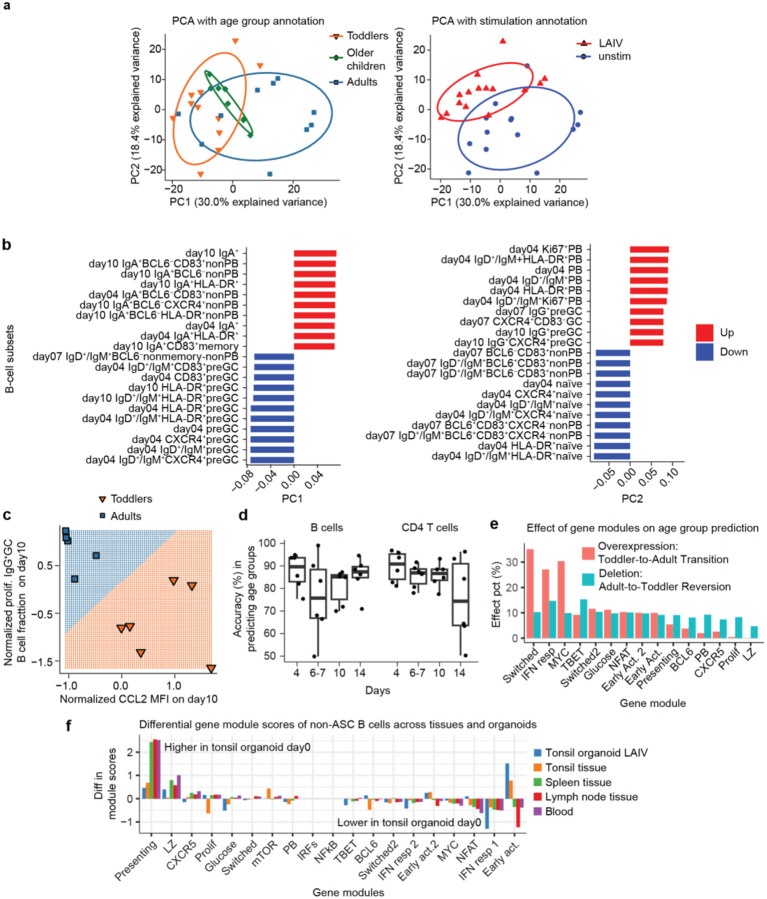
Age dominated the variation in immune responses to LAIV. (**a**) PCA plots displaying the top two variances of flow-measured B-cell subset fractions across all age groups, both with and without LAIV stimulation. (**b**) Bar plots showing the top B-cell subset fractions contributing to the top two principal components. (**c**) Contour plot illustrating that the age groups (toddler and adult groups) were accurately predicted by proliferating IgG^+^GC B cell fraction and CCL2 concentration from the supernatants on day 10. The separation of the feature space was calculated using the Support Vector Machine model. (**d**) Boxplot depicting the accuracy of age group predictions achieved by fine-tuning the scRNA-seq AI foundation model Geneformer with our organoid scRNA-seq dataset. (**e**) Boxplot showing the percentage of single cells with a predicted age-group change in response to either gene module overexpression or deletion calculated in the AI Geneformer model. (**f**) Boxplot depicting the log-fold-change of module scores between untreated (day0) tonsil organoid and LAIV-treated tonsil organoids as well as different healthy tissues for the gene module scores.

Throughout our analysis, we have highlighted the differences in immune responses to LAIV between toddlers and adults. However, the key question remains: how significant is donor age in driving these variations in immune response? To address this, we conducted principal component analysis (PCA) on all the B-cell subset fractions measured by flow cytometry and antibody titers across day 4 to day 10 (Fig. 5a). We found that age was the primary factor influencing variation, as age groups aligned well with the first component in the PCA, even more so than the effects of LAIV stimulation. Other donor-related factors did not appear to contribute to the top variances (Extended Data Fig.6). This age-related variation is primarily due to differences in IgA^+^B cell subsets and preGC subsets, with IgA^+^B cells predominating in adults and preGC subsets in toddlers (Fig. 5b). This suggests that adults exhibit a more pronounced class-switched mucosal response, whereas in toddlers, B-cell responses are predominantly stalled at the preGC stage, limiting their progression to GC and PB responses.

To identify features that best predicted the distinction between toddlers and adults, we examined multi-omic variables, including B-cell subset fractions, supernatant cytokine concentrations, and antibody titers. We used the LASSO (Least Absolute Shrinkage and Selection Operator) method combined with the leave-one-out cross-validation. Our analysis identified the day 10 proliferative IgG^+^GC B cell fraction and CCL2 cytokine concentration as the two most predictive features in our dataset (Fig. 5c). These features not only appeared most frequently in the LASSO predictive models, but also showed the highest statistical significance in separate t-tests comparing the two age groups. Notably, CCL2 has been associated with influenza infection and the severity of pathology in children^13^, suggesting that the strong CCL2 secretion signature observed in toddlers (Fig. 2c) may indicate a common, potentially pathogenic immune response in young children after flu vaccine or infection that needs to be addressed. In the leave-one-out cross-validation, these predictors consistently identified the correct age groups of all 11 donors in our dataset, achieving an area under the curve (AUC) of 1. Additionally, other features, such as CD83^+^naïve, proliferative IgA^+^GC, and IgA^+^extrafollicular B cells on day10, also demonstrated high predictive accuracy in distinguishing toddler and adult age groups (data not shown).

Previous donor-level analyses may overlook variations across single-cell phenotypes, whereas recent advances in AI foundation models have enabled deeper insights into heterogeneous single-cell transcriptomic data. In our study, we investigated the extent to which age-dependent differences could be captured and predicted at the single-cell level using our dataset. Leveraging the scRNA-seq-based AI model Geneformer with finetuning, we identified age-dependent variations in cellular phenotypes through AI-captured non-linear gene networks. We successfully predicted age group among thousands of cells with accuracies ranging from 76.1% to 89.7% at various timepoints following LAIV stimulation (Fig. 5d). These predictions were robust across multiple timepoints and cell types in both B cells to T cells. Notably, we observed greater prediction variance in B cells around day 7 and in T cells around day 14, suggesting increased complexity or transitional immune dynamics at these stages.

We further performed in-silico gene module perturbations by simulating overexpression or silencing of each gene module. Strikingly, the modules predicted to promote toddler cells toward a more adult-like state overlapped with those identified earlier, including the *TACI*-associated switch module, *TLR7/MYC* module, and TBET module (Fig. 5e). These findings lay an important foundation for further AI-integrated analysis using our dataset as a ‘virtual cell’ platform to model age-dependent immune responses and identify candidate genes driving immunological differences between toddlers and adults.

Additionally, we compared our organoid datasets and public datasets from healthy tissues and blood. We found that tonsil organoids exhibit tissue-specific transcriptional profiles that closely resemble tonsil tissues and are distinct from blood (Extended Data Fig. 6b), supporting the tonsil organoid as a representative in vitro model. In details, tonsil tissues and organoids showed higher abundance of activated, preGC and GC B cells compared with other tissues and blood (Extended Data Fig 6c). Differential gene-module score analysis further revealed that both tonsil tissues and organoids are enriched for antigen presenting module, GC-related modules, proliferation module, and glucose module, highlighting antigen presentation, GC, and active clonal expansion in tonsils relative to other tissues and blood (Fig. 5f). Overall, these findings demonstrate that tonsil organoids closely mirror tonsil tissue at the transcriptional level, validating them as a robust in vitro module for studying tonsil-specific immune responses.

## Discussion

While we have known that young children are very vulnerable to infectious diseases, there is very little specific information as why this is. This is because the data we have had until now has been limited to small blood volumes and the difficulties of longitudinal studies. Here we took advantage of the availability of a key organ in the mucosal immune system of human beings, tonsils, which are routinely removed for sleep apnea across a very broad age spectrum (2–70+ years) and contain billions of lymphocytes. Most importantly the ability to study vaccination responses in this organoid system^2^ gives us a unique window with which to ask how the human immune system develops at different ages. Our results show that toddlers exhibit reduced protection following LAIV stimulation compared to adults, with lower IgA-mediated mucosal and IgG-mediated systemic, B-cell-mediated humoral and cytotoxic-CD8-mediated cellular activity. Specifically, toddlers had lower levels of influenza-specific IgA and IgG antibodies, along with reductions in key follicular and extrafollicular B cell populations. Activated cytotoxic CD8^+^T cells were also less abundant in toddlers. Surprenant analysis revealed diminished CSR-associated cytokine activity in toddlers, including FLT3L, IL17, IL2, and TACI, potentially linked to weaker CD4^+^T-B cell interactions and fewer follicular gdT cells. In contrast, toddlers produced higher levels of pro-inflammatory cytokines--such as CCL2 and PAI1--associated with more severe influenza infection^13,14^. To better understand these differences, we investigated gene regulatory networks and identified critical players involved in immune activation and cell differentiation including TLR and T-bet signaling. Additionally, metabolic analysis showed reduced glycolysis and oxidative phosphorylation in toddlers at multiple timepoints, suggesting an age-dependent metabolic disadvantage that may impact immune functions. Finally, machine learning analysis of our multi-omic dataset identified age as the dominant driver of variation in immune responses among donors tested for the influenza vaccine and key predictors including cytokine CCL2 and switched GC responses on later days best distinguishing immune responses among these groups. These findings help to fill out a more comprehensive understanding of age-dependent vulnerabilities in immune responses, highlighting potential therapeutic targets and vaccine strategies specific to young children.

Previous studies in mice and humans have shown that influenza-specific IgA antibodies predominantly protect the upper respiratory mucosa, whereas IgG antibodies are mainly found in serum and serous fluid in lungs, playing distinct roles in protecting against mucosal infection and viral pneumonia, respectively^40^. Some research has linked IgA deficiency in patients to variants in the *TNFRSF13B* gene^15^, encoding the TACI protein. Consistently, our observations corroborate past research, showing fewer TACI transcripts and secretory protein in toddlers, which correlate with reduced IgA-mediated mucosal immunity, and can contribute to impaired T-independent responses^16^. Whether this deficiency results from developmental layers of functionally distinct cell subsets^41,42^ remains to be determined. Additionally, while T-bet signaling in B cells is known to promote IgG CSR and PB differentiation in response to flu infection^43,44^, its potential role in IgA CSR remains unclear. Although IgA deficiency can sometimes be compensated for by increased systemic IgG, cytokines, and activated CD4 and CD8 T cells^15^, we observed only increased proportion of activated CD4 T cells and elevated levels of pro-inflammatory cytokines induced by influenza infections in toddlers. Future investigations should explore the mechanistic distinction between mucosal and systemic immunity and identify the causal and compensatory factors involved.

In our initial report, we found that tonsil organoids from children under five lacked T-independent responses to LAIV^2^. Here, we identified potential signaling cues that may underlie this limitation, including reduced TLR signaling, TACI expression, and IgG2^+^PB levels. Notably, we did not observe evidence of impaired BCR crosslinking signals, suggesting TLR impairment as the sole limitation in upstream antigen recognition of influenza TI antigens^4^. These T-independent responses can be crucial for mucosal immunity due to their rapid activation and ability to drive class switching. Considering the link between IgA and TACI^15^ and the essential role of TACI in TI responses^45^, IgA-mediated mucosal immunity may also play a pivotal role in TI responses, though this has not been extensively investigated^46,47^. In mice, the weaning period occurs almost immediately before puberty^48,49^, making it particularly challenging to longitudinally study stimulation-dependent early-life immune responses in the absence of maternal antibodies, which have a potential suppressive effect on both T- independent^24,50^ and T-dependent^51^ responses. Therefore, our human organoid model demonstrates a unique advantage for studying T-independent responses longitudinally, particularly in younger children.

In our age-based comparison of the LAIV vaccine, we did not distinguish between naïve and memory immune responses. A lack of prior exposure remains a critical factor contributing to young children’s high susceptibility to most pathogens, which must be carefully considered in vaccine design for this population. Our study focused on the combined effects of immune responses rather than isolating naïve and memory contributions. Future research specifically aims at naïve vaccine development could provide deeper insights into this important aspect. Additionally, environmental exposures beyond influenza vaccines play significant roles in shaping immune development among young children, influencing distinct cellular and cytokine profiles^52^. Children raised in less hygiene environments tend to exhibit more functional CD8 and gdT cells, higher CCL4 levels and less IL8 levels^52^—features that resemble adult-like immune responses in our organoids. Recent studies have also shown that microbiota composition follows a strongly age-dependent trajectory, further impacting immune function and disease susceptibility^53–55^. For example, it is not clear whether the suppression of Th17 cytokine in infants due to *Bifidobacterium* enrichment^56,57^ and limited IL17 cytokine we observed in toddler organoids are associated. Despite the timing of gut microbiome maturation is still debated^54^, further research aligning microbiota and immune development will be essential to better understand their interactions and advance pediatric health strategies.

Our age-related study focused on tonsil organoids as a model representing mucosa-associated lymphoid tissues and aimed to address tissue-specific responses, particularly in the context of vaccine-driven immune activation. Previous research has identified age-related immune differences in both peripheral blood and other tissues, including myeloid cells, gdT cells, T_reg_ cells, and tissue resident cells, many of which function as regulatory mechanisms^23,28,37,58–63^. For instance, systemic serum IgG antibodies remained stable in adults over 7 months, contrasting with children immunized with IIV who experienceda 4-fold decrease in antibody titers over a similar period^64^. In our study, we found that mucosa-associated IgA-promoting immune factors and their chemokine receptor and integrin expression, which are crucial for migration to HEVs and lungs, were limited in children. These findings are critical for vaccine design targeting distant mucosal tissues. Appropriate cytokine cues can even tune and program the development of immune responses in early life^42,61^. We observed that adult tonsil organoids secreted more than twice the amount of FLT3L cytokine compared to toddlers, consistent with previous studies suggesting FLT3L as an adjuvant for nasal administration to enhance mucosal responses^65^. Our model provides a unique mucosal and lymphoid tissue perspective, offering longitudinal data that was largely missing in previous blood studies. Ongoing studies are testing new vaccines and adjuvants in human tonsil organoids, and future work can help better address agreements and distinctions between mucosal and systemic models.

## Methods

### Human Tonsil Tissue Collection and Organoid Preparation

Whole tonsils were obtained as discarded tissue from pediatric and adult donors undergoing tonsillectomy for obstructive sleep apnea or hypertrophy at Stanford Hospital. All samples were collected in accordance with the Stanford University Institutional Review Board (Stanford IRB-60741, IRB-30837). Written informed consent was signed by all adult participants or legally authorized representatives for pediatric participants.

Tonsil samples were processed as previously described^2,66,67^. Briefly, whole tonsil samples were collected immediately after surgery and incubated in Ham’s F-12 medium (ThermoFisher, cat #31765092) containing Normocin (Fisher, cat #NC9273499) Penicillin/Streptomycin/Amphotericin B (ThermoFisher, cat #15240062) for at least 30min at 4 °C before processing. The tonsils were then manually minced into small pieces (~5 mm × 5 mm × 5 mm) and passed through a 100 um filter using a syringe plunger to obtain a cell suspension. The filter was washed repeatedly with complete immune organoid medium (RPMI with GlutaMAX, 10% FBS, 1X Penicillin/Streptomycin/Amphotericin B, 1mL Normocin, 5mL Insulin/transferrin/selenium supplement (ThermoFisher, cat #41400045), 1X non-essential amino acids, 1X sodium pyruvate). Debris was removed through Ficoll density gradient separation. The collected cells were washed with PBS, counted, and cryopreserved in FBS with 10% DMSO. Frozen aliquots were stored at −180 °C until use.

### Human Tonsil Organoid Culture and Treatment

For tonsil organoid preparation, cryopreserved single-cell tonsil aliquots were thawed and re-suspended at 6 × 10^7^ cells/mL in complete immune organoid medium. 12-well tissue culture plates were prepared with 0.4 um pore size permeable membrane transwells (24-well size PTFE or polycarbonate membranes, Millipore, cat #PICM01250) placed into each well. A 100 uL cell suspension (6×10^6^ cells) was plated into each transwell. 1 mL of complete immune organoid medium containing 0.5 ug/mL BAFF (Biolegend, cat #559608) was added to the outer well. 1 uL of LAIV (around 10^4−5^ fluorescent focus units per strain; FluMist Quadrivalent, MedImmune) was added directly to each well for stimulation during culture setup. If needed, an CD154 inhibitor, anti-CD154 antibody (Biolegend, cat #310828) was added to the cell-containing proportion at a final concentration of 10ug/mL at the start of the culture. Tonsil organoids were supplemented every 3–4 days with 500 uL of complete immune organoid medium containing 0.5 μg/mL BAFF. Organoids were incubated for 4–14 days at 37 °C, 5% CO2 with humidity and were harvested for further analysis. Supernatants were collected during cell harvesting and stored at −20°C or −80°C for later profiling.

For OXPHOS inhibition, Oligomycin A (MilliporeSigma, cat #75351) was added to the culture medium at a final concentration of 10 uM for 12hr. Metabolite uptake experiments were conducted using glucose-free medium (ThermoFisher, cat #11879020) with 50 uM 2NBDG (ThermoFisher, cat #N13195) or 0.1 uM BODIPY FL C_12_ (ThermoFisher, cat #D3822) for 25-min treatments. Following these treatments, cells were harvested for flow cytometry analysis.

### Flow cytometry

Cells from organoids were stained using Human TruStain FcX (Biolegend, cat #422302), Zombie Aqua^™^ Fixable Viability Kit (Biolegend, cat #423102), Brilliant Violet staining buffer (BD, cat #566349) and a panel of surface markers, including CD45 (Biolegend, clone HI30), CD3 (Biolegend, clone OKT3), CD19 (clone 4G7, Biolegend; clone HIB19, BD), TCRab (Biolegend, clone IP26), TCRgd (Biolegend, clone B1), CD4 (BD, clone OKT4), CD8 (Biolegend, clone RPA-T8), CD83 (Biolegend, clone HB15e), CXCR4 (BD, clone 12G5), IgG (Biolegend, clone 1310G05), IgD (Biolegend, clone IA6–2), IgM (Biolegend, clone MHM-88), IgA (Miltenyi Biotec, clone IS11–8E10), HLA-DR (Biolegend, clone L243), CD38 (Biolegend, clone HB-7), CD27 (Biolegend, clone O323), followed by analysis via flow cytometry. Intracellular staining was performed using the FOXP3 staining set (ThermoFisher, cat #00-5523-00) and included intracellular markers such as Ki67 (Biolegend, clone Ki-67), BCL6 (BD, clone K112–91), and AID (BD, clone EK2–5G9).

### ELISA assays

Influenza-specific antibodies were detected using ELISA assays as previously described^2,66,67^. High-binding assay plates (Corning) were coated overnight with season-matched Fluzone Quadrivalent inactive influenza virus at a final concentration of 2 ug/mL in sodium carbonate/bicarbonate ELISA coating buffer. Plates were blocked with 1% BSA in PBS for 2 hours at room temperature (RT). Culture supernatants were serially diluted from 1:1 to 1:300 in PBS and added to coated and blocked plates for 1 hour at RT. Horseradish peroxidase (HRP)-conjugated anti-human secondary antibodies to polyvalent IgM/IgG/IgA (Abcam, 1:5,000 dilution), polyclonal IgA alone (SouthernBiotech, cat #2020–05, 1:12,000 dilution), IgG (SouthernBiotech, cat #2040–05, 1:50,000 diluation), or IgM (SouthernBiotech, cat #2020–05,1:12,000 dilution) were used to detection. Plates were developed with TMB substrate solution (Thermo Scientific), quenched with sulfuric acid, and read at 450 nm.

For cytokine detection in supernatants, including BAFF, APRIL, TGF-b1 and secreted cytokine receptors TACI and BCMA, customized ELISA kits were used according to the manufacturer’s instructions without supernatant dilutions (BAFF: abcam, cat #ab119579; APRIL: ThermoFisher, cat #BMS2008, and AdipoGen Life Sciences, cat #AG-45B-0012-KI01; TGF-b1: ThermoFisher, cat #BMS249–4;TACI: R&D Systems, cat #DY174; BCMA: R&D Systems, cat #DY193).

### Luminex Assay

The Luminex assay was performed by the Human Immune Monitoring Center at Stanford University-Immunoassay Team. The EMD Millipore Human 80 Plex kits (EMD Millipore Corporation, Burlington, MA) included 3 panels: Panel 1 (Milliplex HCYTA-60K-PX48), Panel 2 (Milliplex HCP2MAG-62K-PX23), and Panel 3 (including Milliplex HSP1MAG-63K and HADCYMAG-61K-03 for Resistin, Leptin, and HGF, forming a 9 plex). The assay followed the manufacturer’s recommendations with the following modifications: Briefly, 25ul of undiluted supernatant sample was mixed with antibody-linked magnetic beads in a 96-well plate and incubated overnight at 4°C on an orbital shaker at 500–600 rpm. Plates were washed twice with wash buffer using a BioTek ELx405 washer (BioTek Instruments, Winooski, VT). The samples were then incubated at room temperature for one hour with 25 uL of biotinylated detection antibody per well, followed by the addition of 25 uL of streptavidin-PE for 30 minutes with shaking. After washing, 1X reading buffer was added to the wells for analysis on the Luminex FlexMap3D Instrument, with a lower bound of 50 beads per sample per cytokine. Each sample was measured in duplicate. Custom Assay Chex control beads (Radix BioSolutions, Georgetown, Texas) were included in all wells. Wells with bead counts below 50 were flagged, and data from wells with bead counts below 20 were excluded from analysis.

### BD Rhapsody targeted scRNA-seq

Tonsil organoid cells were harvested, FACS-sorted, library prepared and sequenced as follows. Cells were initially stained with oligonucleotide-conjugated Sample Tags (BD Human Single-Cell Multiplexing Kit, cat #633781), LIVE/DEAD Aqua Zombie stain, and minimal surface markers for identifying B and T cells. Live B and T cells were then FACS-sorted from the barcoded samples and pooled. The pooled sample was subsequently stained with customized oligonucleotide-conjugated antibodies for the Antibody-Seq (Ab-seq) Stain in BD stain buffer for 30 min on ice. Samples were spun down at 350*g* for 10 min and washed three times before the pellet was resuspended in Rhapsody Sample Buffer for cell capture. Cell capture and library preparation were performed using the BD Rhapsody Targeted mRNA and AbSeq Reagent Kit (cat #633774). Briefly, cells were captured with beads in a microwell plate, followed by cell lysis, bead retrieval, cDNA synthesis, template switching, Klenow extension, and library preparation, all conducted at the Stanford Human Immune Monitoring Centre following the BD Rhapsody protocol. Libraries were prepared for sample tags, AbSeq and targeted mRNA using the customized Immune Response panel. Sequencing was conducted on a NovaSeq (Illumina) at Novogene USA.

### ScRNA-seq Analysis

The Rhapsody scRNA-seq and CITE-seq raw data were initially preprocessed using the Seven Bridges Genomics online platform, employing the BD Rhapsody Targeted Analysis Pipeline to align genes and calculate molecular counts with unique molecular identifier (UMI) distribution-based error correction (DBEC). After preprocessing, the single-cell Rhapsody count tables were imported in Seurat package^68^ in R (v.4.3.1) for further analysis. Both gene and protein counts were log1p-transformed and integrated across batches using canonical correlation analysis. The combined genomic and proteomic data were scaled, regressing out cell cycle gene effects. Dimension reduction was performed on this combined dataset using principal component analysis with 30 dimensions. Graph-based clustering and manual annotation were applied to identify cell subsets. To identify DEGs between two age groups, Wilcoxon rank-sum tests were conducted on the log-normalized genomic and centered-log-ratio-transformed proteomic data. These tests were repeated 50 times by bootstrapping, using either the minimal or geometrical mean number of cells from each age group across various timepoints to ensure consistent comparisons. *P*-values were adjusted using the Benjamini-Hochberg correction method across all timepoints and bootstraps. For each marker and timepoint, we calculated a geometric mean of adjusted P-values and a mean of log2 fold change in normalized expression for DEG output.

For the trajectory analysis we used cytoskel^69^. For the regulon specificity score (RSS) and network analysis of the B cells subset, the Seurat objects were converted to loom files in R (V4.2.1) using the ScopeLoomR package (V0.13.0). The subsequent RSS analysis and network analysis were conducted using the pySCENIC (V0.12.1)^19^ and networkx (V3.2.1) packages in Python (V3.10.14). Gene modules were identified by hierarchical clustering based on the correlation matrix of the log-normalized genomic and proteomic counts. To avoid imputation bias, only genes and the proteins sequenced in all donors were included. The optimal number of gene modules was manually selected to ensure critical functional genes were grouped appropriately, including those related to proliferation, type I IFN, glycolysis, GC activities and immune cell activation. We used the AddModuleScore function from Seurat^68^ to calculate the associated gene module scores. Partial correlation network among gene module scores were calculated using bootnet^70^. The partial correlation values were compared using the permutation tests by NetworkComparisonTest (V2.2.2) and the significance was adjusted by FDR method.

## Supplementary Material

Supplementary Files

This is a list of supplementary files associated with this preprint. Click to download.

- DAVISreportingsummary.pdf
- SunetalSuppTable1.xlsx
- SunetalSuppTable2.xlsx
- SunetalSuppTable3.xlsx
- SunetalSuppTable4.xlsx
- SunetalExtendedDatasubmission.docx
